# Packages of Care for Alcohol Use Disorders in Low- And Middle-Income Countries

**DOI:** 10.1371/journal.pmed.1000170

**Published:** 2009-10-27

**Authors:** Vivek Benegal, Prabhat K. Chand, Isidore S. Obot

**Affiliations:** 1 Deaddiction Centre, National Institute of Mental Health and Neurosciences, Bangalore, India; 2 University of Uyo, Uyo, Nigeria; 3 Centre for Research and Information on Substance Abuse (CRISA), Uyo, Nigeria; London School of Hygiene and Tropical Medicine, United Kingdom

## Abstract

In the fourth in a series of six articles on packages of care for mental disorders in low- and middle-income countries, Vivek Benegal and colleagues discuss the treatment of alcohol use disorders.

*This is the fourth in a series of articles highlighting the delivery of “packages of care” for mental health disorders in low- and middle-income countries. Packages of care are combinations of treatments aimed at improving the recognition and management of conditions to achieve optimal outcomes.*

**Summary Points**

- Alcohol use disorders (AUDs)—conditions that range from hazardous and harmful alcohol use to alcohol dependence—are a low priority in low- and middle-income countries (LMICs), despite causing a large health burden.
- Most alcohol-related harm is attributable to hazardous/harmful drinkers who make disproportionate use of primary health care systems, but often go undetected and untreated for long periods, even though brief, easily delivered interventions are effective in this group of people.
- Health care systems in LMICs currently focus on providing tertiary care services for the treatment of dependence (where there is often a poor outcome). This focus needs to shift towards the cost-effective strategy of providing brief interventions for early AUDs.
- Effective evidence-based combinations of psychosocial and pharmacological treatments for AUDs are available in LMICs but are costly to implement. Policy makers need to ensure that people with AUDs are offered the most appropriate services using stepped-care solutions that start with simple, structured advice for risky drinkers and progress to specialist treatment services for more serious AUDs.
- LMICs also need to improve their implementation of proven population-level preventive measures to reduce the health burden due to AUDs. An international Framework Convention on Alcohol Control may help them do this.

## Introduction

Alcohol misuse is responsible for a disproportionately high health burden, accounting for almost 5% of all ill health and premature death worldwide in 2004. The impact of alcohol misuse is worst among poor populations and in low- and middle-income countries (LMICs) where the disease burden per liter of alcohol consumed is greater than in wealthy populations. In 2004, the western Pacific region, Southeast Asia, and the Americas had the highest prevalence of alcohol use disorders (AUDs) relative to the average volumes of alcohol consumed. Alcohol attributable net disability adjusted life years (DALYs) were 13,406, 7,343, and 3,392 in China, India, and Brazil, respectively, and 594 and 393 in Germany and Japan, respectively, in the same year [1]–[7].

Sustained, heavy alcohol exposure leads to a chronic relapsing illness with a characteristic syndromal presentation termed “dependence.” However, alcohol misuse can produce harm without the presence of dependence. The terms “hazardous drinking” and “harmful drinking” describe patterns of use likely to result in or having resulted in physical/psychological harm, respectively, without satisfying the International Classification of Disease (ICD) ten criteria for alcohol dependence (Box 1) [8],[9].

**Box 1. International Classification of Disease 10 Criteria for AUDs**

**Hazardous use** [ICD 10; Z72.1]

“A pattern of alcohol consumption that carries with it a risk of harmful consequences to the drinker. These consequences may be damage to physical or mental health, or social consequences to the drinker or others. Other potential consequences include worsening of existing medical conditions or psychiatric illnesses, injuries caused to self or others due to impaired judgment after drinking, high risk sexual behaviors while intoxicated, and worsening of personal or social interactions”. Hazardous use is often operationalized as an average consumption of 21 drinks or more per week for men and 14 drinks or more per week for women. It is recognized by the World Health Organization (WHO) as a disorder distinct from other AUDs [26].

**Harmful use** [ICD 10; F10.1]

“A pattern of drinking that is already causing damage to health. The damage may be either physical (e.g., liver damage from chronic drinking) or mental (e.g., depressive episodes secondary to drinking).

Harmful patterns of use are often criticized by others and are sometimes associated with adverse social consequences of various kinds. However, the fact that a family or culture disapproves of drinking is not by itself sufficient to justify a diagnosis of harmful use” [9],[26].

**Alcohol dependence** [ICD 10; F10.2]

“A cluster of behavioural, cognitive, and physiological phenomena that develop after repeated alcohol use and that typically include a strong desire to take alcohol, difficulties in controlling its use, persisting in its use despite harmful consequences, a higher priority given to alcohol use than to other activities and obligations, increased tolerance, and sometimes a physical withdrawal state” [9].

Most alcohol-related harm is attributable to hazardous/harmful drinkers rather than to people with alcohol dependence [10]–[12]. However, this distinction is rarely made, especially in LMICs where politicians, planners, and the public discourse have focused primarily on alcohol dependence—the conventional central motif of alcohol misuse. Concentrating on the rarer presentation of dependence only serves to minimize the problem, stigmatize the condition, and marginalize affected individuals.

Studies of alcohol treatment systems across countries show that the size, extent, and character of the treatment system each country adopts depends more on its view of the importance of alcohol problems (and its reliance on alcohol excise) than on actual changes in alcohol consumption achieved by the system, the need for treatment in the country, or the economic resources available for treatment [13]. Furthermore, recent reviews of the current situation in LMICs indicate that service systems for the treatment of AUDs, where available, are mainly oriented to tertiary treatment of dependence with an emphasis on long-term residential treatment in rehabilitation centres, specialised clinics, or psychiatric hospitals [14]–[16]. These facilities are mainly concentrated in urban areas and are often in private settings, usually with high fee structures. Where government-funded treatment/counseling centers are available, the overall efficacy of these programmes is low [17]. Consequently, many people with AUDs in LMICs remain untreated (the median treatment gap for AUDs in LMICs is 78.1%) because they first seek help for early alcohol-related problems from primary health care providers who are not trained to recognise the problem [18]–[20]. Those who are finally treated often have to wait for over a decade before receiving treatment for their alcohol misuse [21]. Thus, in many LMICs, alcohol-related problems are first addressed when they are already severe and difficult to treat, secondary prevention in earlier stages of drinking problems is virtually nonexistent, and many heavy drinkers who are at risk of developing AUD in the future are not targeted by health interventions.

In this article, we focus on the effective management of AUDs in LMICs. We review the available evidence on the efficacy of treatments and the delivery of interventions derived from LMICs. Because that evidence is often limited, we also cite systematic reviews and meta-analyses based on global evidence and key randomized controlled trials (RCTs) from high income countries (HICs) where appropriate. On the basis of our review, we propose a package of care—a combination of treatments aimed at improving the recognition and management of conditions to achieve optimal outcomes—for AUDs.

## The Evidence on the Treatment of AUDs

Although there is now a substantial evidence base about the relative effectiveness of different strategies for reducing the rates of alcohol-related harm, most of the evidence derives from HICs and cannot be transposed directly to LMIC settings. In Table 1, we review the existing data and in this section, we discuss some aspects of the evidence base in more detail.

**Table 1:** Evidence in support of treatments for AUDs.

Intervention	Evidence from HIC	Evidence from LMIC
**Early detection/screening**	Systematic reviews and RCTs of screening tools (AUDIT, CAGE, and RAPS4) for alcohol problems in primary health care and other health care settings [23]–[26]	Validation of AUDIT in LMICs [27]–[29]
**Brief intervention**	Meta-analyses of brief interventions [30]–[36]	RCTs of brief interventions in Brazil, India, and Taiwan [37]–[39]
	Systematic review of effects of a combination of educational and office support programs on rates of screening and advice giving by primary health care providers [40]	—
**Psychosocial therapies for relapse prevention**		
Structured interventions	Meta-analyses and systematic reviews of structured interventions [42]–[45]	RCT of culturally modified cognitive behavioral treatment in dependent drinkers in Korea [46]
Alcoholics Anonymous and other self-help groups	Cochrane review of Alcoholics Anonymous and Twelve Step Facilitation approaches for reducing alcohol dependence or problems [47]	—
**Pharmacotherapy in detoxification and relapse prevention**		
Benzodiazepines	Cochrane review of benzodiazepines for alcohol withdrawal [48]	RCT comparing of lorazepam and chlordiazepoxide for alcohol withdrawal in India [49]
Disulfiram	Multisite RCT in the US [50]; RCT in Finland [94]	RCT in India [69]
Naltrexone (opiate antagonist)	Meta-analyses, a Cochrane review, and several RCTS of oral naltrexone and intramuscular depot forms of naltrexone [53]–[57],[60]	RCT in Taiwanese Han males [58]; RCT in Iran [59]
Acamprosate (glutamate inhibitor)	Three meta-analyses [62],[63],[66]; two large RCTs including the US COMBINE trial [59],[64]	Multicenter RCT in combination with out-patient psychosocial intervention in Korean alcohol-dependent patients [65]
Topiramate (glutamate antagonist	Two RCTs [67],[68]	RCT comparing disulfiram and topiramate in patients with alcohol dependence in India [69]
Baclofen (GABA receptor agonist)	Two RCTs investigating baclofen for maintenance of abstinence and its safety patients with liver cirrhosis [70],[71]	—

### Population-Level Interventions for Prevention

For populations with high rates of hazardous alcohol use, both population-wide measures (for example, taxation on alcoholic beverages) and individual-based interventions (for example, brief physician advice) have been shown to have a notable impact on reducing the global burden of alcohol misuse, although higher rates of taxation may be ineffective in countries with undocumented consumption. Other population-wide strategies, such as reduced hours of sale and advertising bans, seem to have less impact. For populations with low rates of hazardous drinking, intervention strategies targeted at particular subgroups of the drinking population, such as drivers who drink or primary-care attendees with already high levels of alcohol consumption, appear to be more cost effective than population-wide strategies like taxation [12],[22].

### Early Detection and Brief Interventions for Early AUDs

The treatment gap in LMICs can be narrowed by broadening the base of treatment and by opportunistic screening and brief intervention (SBI) in primary health care settings. Several brief screening instruments have been developed in HICs and one of these—AUDIT (Alcohol Use Disorders Identification Test)—has been shown to provide an accurate measure of risk across gender, age, and cultures in several LMICs [23]–[29]. SBI provides a framework for stepped intervention to help risky drinkers reduce or stop alcohol consumption, which starts with simple but structured advice, progresses to extended brief interventions, and ends with referral to a specialist alcohol treatment service for those with more serious problems and those who fail to respond to brief interventions. Several reviews and meta-analyses of research evidence collected in HICs in a variety of health care settings and three RCTs in LMICs have concluded that SBI can effectively reduce alcohol consumption to low-risk levels among hazardous and harmful drinkers [30]–[39]. Other evidence from HICs suggests that a combination of office support programs and education of primary health care providers can increase the rate of screening and advice giving of primary-health care providers [40]. Finally, there is evidence from HICs that SBI can decrease alcohol-related mortality for up to 16 y after the intervention [41].

### Psychosocial Interventions to Prevent/Delay Relapse

These interventions fall into two main categories: structured interventions and self-help groups. Two large US- and UK-based RCTs that compared psychosocial therapies differing widely in conceptual framework, intensity, duration, and location (Motivation Enhancement Therapy [MET], Cognitive Behavior Therapy [CBT], Twelve Step Facilitation [TSF] therapy, and Social Behavior and Network Therapy [SBNT]) found minimal long-term difference between inpatient/residential treatment and outpatient counseling approaches [42],[43]. These trials also found approximately equivalent (and reasonably good) outcomes with both brief, nonintensive treatments (MET) and intensive treatments (CBT, TSF, and SBNT) for moderately severe alcoholics. A systematic review that considered evidence collected in HICs concluded that manual-guided specific treatments with a theoretical base (e.g., MET, CBT) are better than nonspecific treatments (supportive therapy and social work interventions), but that among the specific therapies none was superior [44]. The same review found that marital therapy and family intervention yielded positive results. A meta-analysis of behavioral self-control training found that this intervention reduced alcohol consumption and alcohol-related difficulties [45]. Very few studies have examined psychosocial interventions in LMICs, but one RCT in dependent drinkers in Korea found that culturally modified cognitive behavioral therapy increased the drinkers' insight into their condition [46].

A Cochrane review of studies investigating the effectiveness of strategies adopted by Alcoholics Anonymous and other self-help groups to reduce alcohol dependence provided no definitive evidence that these approaches are effective in HICs; there are no data from LMICs about the effectiveness of self-help groups [47].

### Pharmacotherapy for Detoxification and Relapse Prevention

Conventionally, pharmacotherapy involves the use of benzodiazepines for detoxification and disulfiram for relapse prevention. A systematic review from HICs showed that benzodiazepines remain the agents of choice for treating alcohol withdrawal during detoxification [48]. A recent RCT from India that compared lorazepam and chlordiazepoxide found that these benzodiazepines had comparable attenuating effects on uncomplicated withdrawal [49]. Thus, lorazepam can be used in LMIC settings where it is difficult to test liver function status, an essential preamble to using long-acting benzodiazepines in patients.

A multisite RCT from the US concluded that the aversive agent disulfiram might help prevent relapse in compliant patients but is ineffective at promoting continuous abstinence [50]. Outcomes were improved, however, if a supportive family member was able to monitor compliance. RCTs undertaken in LMICs where disulfiram is still the most commonly used medication for AUDs because it is cheap and easily available show that it continues to be a useful treatment particularly when compliance with the drug regimen is overseen by family members [51].

A recent systematic review of data from HICs provides substantial evidence that newer agents such as naltrexone, acamprosate, topiramate, and baclofen have modest effects on improving most outcome indicators (abstinent days, heavy drinking days, days to lapse/relapse, and work and social functioning) in alcohol-dependent individuals, although they do not guarantee abstinence. Furthermore, when accompanied by brief advice, these agents have been shown to improve overall outcome [52]. Although these newer agents are relatively costly (which limits their use in LMICs), they nevertheless offer a paradigm shift in the treatment of AUDs. Treatment with these agents can be initiated while an individual is still drinking heavily and at the point of maximum crisis and help-seeking. They can also be safely delivered in general practice and many other health care settings (unlike the scheduled drug disulfiram, which, because of its toxicity and propensity to cause severe reactions with alcohol, had to be strictly monitored and could only be prescribed by addiction specialists), thus broadening access to treatment. Although abstinence remains the ultimate goal in treating alcohol-dependent individuals, reducing the frequency of heavy drinking has the major impact of decreasing alcohol-related consequences and improving quality of life. These agents may also support effective treatment of hazardous/harmful alcohol use in primary health care settings [53].

Two systematic reviews and several RCTs that have investigated the use of the opiate antagonist naltrexone for preventing relapse in both HICs [54]–[57] and LMICs [58],[59] have suggested that this drug reduces the risk of relapse among recently abstinent, alcohol-dependent individuals, though the effect-size is small. The efficacy of naltrexone is greatest in people with high compliance, in those reporting high levels of craving, and in those with a family history of AUDs [60],[61].

Three large HIC-based meta-analyses of the glutamate inhibitor acamprosate reported an increased percentage of nonheavy drinking days and increased continuous abstinence rates at 6 mo when compared to placebo but the effect sizes were small [62]–[64]. However, both the multisite US COMBINE trial [57] and an RCT of acamprosate combined with out-patient psychosocial intervention in Korean alcohol-dependent individuals [65] failed to find any therapeutic benefit for this agent. A meta-analysis of studies that compared the efficacy profiles of naltrexone and acamprosate concluded that acamprosate was likely to be more effective in preventing a lapse, whereas naltrexone more effectively prevented a lapse becoming a relapse [66].

Table 1 also provides details of RCTs that have examined the effect of the glutamate receptor antagonist topiramate (in HICs and in India) on abstinence, relapse, and other alcohol-related outcomes [67]– and two RCTs that have examined the effect of the GABA-B receptor agonist baclofen on drinking outcomes in HICs [70],[71].

## Delivery of Effective Interventions

Despite accruing evidence that medications may support effective treatment of AUDs, treatment systems in LMICs by and large continue to be dominated by psychosocial or religious models and self-help groups that generally disavow biomedical interventions [72],[73]. There is also often limited availability of these drugs in developing countries, their prices are high in the open market, and public-health systems do not supply or subsidize these medications. This last barrier to drug treatment for AUDs in LMICs is not surprising given the unacceptably low spending on health in these countries [74]. Furthermore health insurance is not readily accessible in LMICs, and even if it were present, AUDs are rarely covered by health insurance [75]. In Table 2, we propose a series of steps that might be taken to improve the delivery of care for AUDs in LMICs and in the rest of this section we provide a brief discussion of some of these steps.

**Table 2:** Delivering treatments for AUDs.

Step	How	By Whom	In What Settings
**Increasing consumer demand and awareness**	Reduce public stigma; Reduce therapeutic nihilism in health care professionals; Influence policy makers and public opinion; Establish an international alcohol policy initiative.	Addiction medicine specialists, mental health professionals, media personnel; WHO and its member states [93]	Community, primary health care, specialist care
**Reducing the impact of hazardous drinking**	Implement population level preventive strategies using multisectoral approaches [22]	Taxation and civil administrative authorities; Police; Mental health professionals	Community, civil society
**Increasing access and recognition**	Target early problems; Opportunistic screening; Community treatment camps; Integration with other noncommunicable disease delivery systems	Primary health care; Medical specialists Community nurses; Community health workers [76]–[78]	Clinics, hospitals, emergency rooms, community
**Increasing capacity/reducing costs/improving efficiency**	Training in SBI [85],[86]; Training in manual-based psychosocial interventions [90],[91]	Addiction medicine specialists; Mental health specialists	Specialized de-addiction centers, psychiatry, psychology or social work departments
**Initiating evidence-based treatments**	Stepped-care approach [40],[83]	Primary health care personnel, other medical specialties, specialized addiction treatment providers	Clinics, hospitals, emergency rooms; Community-based treatment camps
**Managing serious cases**	Referral to specialist treatment centers	Addiction medicine specialists, psychiatrists and other mental-health professionals	Specialized de-addiction centers, psychiatry wards of general hospitals, rehabilitation centers.
**Achieving optimal recovery/outcome**	Follow up in the community; early referral on relapse	Community health workers, self-help groups (Alcoholics Anonymous)	Primary health care clinics, Alcoholics Anonymous meetings; community
**Addressing impacts of the disorder on other health and social outcomes**	Integration with other noncommunicable disease delivery systems (screening for risk factors), integration with general mental-health delivery systems	Other medical specialists; mental-health specialists; policy planners	Primary health and specialist medical settings, community health camps

### Interventions to Increase Consumer Demand and Awareness

The social stigma attached to AUDs, the lack of knowledge about available treatments, and poor sensitization among primary health care providers delays treatment seeking by people with early AUDs. In turn, because of the poor outcome of advanced AUDs with conventional treatments, some health care professionals believe there is little point in trying to treat people with alcohol problems (therapeutic nihilism). These factors contribute to the large treatment gap in LMICs, and the low demand for services, coupled with the perceived economic “benefits” from alcohol-taxes, fuels official apathy towards upgrading services for the treatment of AUDs [15]. To increase consumer demand and awareness in order to get governments and planners to re-examine the status quo, stakeholders in LMICs should pro-actively disseminate the accruing evidence about the economic and social costs of alcohol misuse in their country. They should also educate their populations about the new understanding of AUDs as a treatable brain disorder and about the availability of effective treatments and use the media to influence the public discourse and to sensitize policy makers and medical professionals [73].

### Interventions to Increase Access to Treatment

In LMICs, detoxification and medical treatment of mild-to-moderate alcohol withdrawal states can be safely managed in out-patient or ambulatory settings under the supervision of community nurses with medical support from local medical practitioners [76]–[80]. Furthermore, there is evidence that a “camp approach” in which patients with substance dependence are treated in brief residential camps in the community is clinically feasible and cost-effective compared to inpatient hospital treatment [81]. Patient access to treatment can also be greatly expanded by combining brief, standardized behavioral treatments with the newer medications. Such combinations improve overall outcome and can be delivered in general practice and other primary health care settings. Importantly, patients with chronic illnesses like AUDs benefit from the continuity of care that primary health care professionals located in the patients' community can provide—heavy drinking and alcohol addiction severity are lower in patient cohorts who receive primary care [82].

### Interventions to Increase the Availability, Reduce the Costs, and Improve the Efficiency of Treatment

A greater proportion of AUDs could be effectively managed with the existing, limited health care resources available in LMICs by incorporating SBI within the normal clinical routine of primary health care doctors and nurses [77]. These practitioners would need to be trained to identify and stage AUDs and to provide a heuristic stepped-care framework of intervention [83]. SBI, which is acceptable to both patients and practitioners, is low-cost and is easily administered by medically trained clinicians with minimal training in AUD treatments [84]. Currently available training methods and manuals for SBI have already been successfully used in LMICs [85],[86].

Integration of interventions for AUDs within existing delivery systems for the care of other noncommunicable diseases would allow effective use of sparse resources. But, since primary health care personnel burdened with multiple responsibilities are often loath to take on additional tasks, AUD treatment services need to be reconceptualised, not as standalone programs, but as part of risk management strategies for other noncommunicable diseases—alcohol misuse constitutes a prominent risk factor for a wide range of noncommunicable diseases. Community level interventions organized in the workplace and by nongovernmental organizations (NGOs) working in areas of development and microfinance also merit further examination [87].

In most LMICs, NGOs and religious/social organizations (mostly unregulated) provide help for AUDs that is often not evidence based and is sometimes even inhumane [88]. The introduction of regulations to ensure minimum standards of care will be difficult because 25%–50% of LMICs do not have a national policy on treatment of AUDs, but should nevertheless be attempted [89].

Several manual-based training schemes that cover psychosocial interventions are freely available on the Internet, but more Web-based resources need to be created and used to increase the availability of treatment for AUDs [90],[91]. Finally, national addiction resource centers where available should be tasked with training, certification, and monitoring, and encouraged to engage in private-public partnerships with available treatment providers.

## Packages of Care for AUDs in LMICs

Although our review suggests that effective measures for combating AUDs exist in LMICs, a degree of scaling down when prescribing care packages for LMIC settings is necessary to reflect the resource availability on the ground. In Table 3 we compare possible packages of care for AUDs in low- and high-resource countries.

**Table 3:** Packages of care for AUDs.

Low Resourced Settings	High Resourced Settings
Opportunistic screening	Opportunistic screening
Stepped-care model, starting with brief advice and working up to extended brief interventions	Stepped-care model, starting with brief advice and working up to extended brief interventions
Community-based treatment of withdrawal (detoxification)	Treatment of withdrawal in community-based or specialized de-addiction/rehabilitation centers
Structured relapse prevention in self-help groups	Structured relapse prevention in self-help groups; specialized interventions including family based interventions
Pharmacotherapy with disulfiram where family monitoring is available and with newer medications where available/affordable	Pharmacotherapy with newer medications
Follow up and monitoring in the community	Follow up and monitoring in the community Follow up and monitoring in specialized centers
Preventive interventions	Preventive interventions

More specifically, on the basis of our review, we propose that the situation in LMICs calls for the adoption of a heuristic stepped-care framework to match the needs of people with AUDs to the most appropriate services. Each step in this framework represents an increased complexity of intervention: (1) step 1 is recognition of alcohol-related problems in primary health care and general hospital settings; (2) step 2 is treatment of hazardous/harmful drinking in primary care; (3) step 3 is treatment of moderate-to-severe dependence in primary health care settings with referrals to specialized units for relapse prevention; (4) step 4 is treatment by mental-health or addiction specialists; (5) step 5 is inpatient treatment. This stepped-framework approach has been used successfully in the treatment of several mental-health problems, notably depression [92], but as yet there are few well-studied models of the approach in the field of substance abuse treatment.

However, it is important to remember that focussing on treatment alone will not reduce the huge burden of disease caused by alcohol in LMICs. Measures that target the drinking environment and the general population in these countries also need to be urgently implemented.

Finally, because the implementation of any of these packages of care or of any other measures to reduce the burden of alcohol-related disease cannot happen in LMICs without the active participation of their governments and of nongovernmental agencies, we strongly support the need for an international health policy initiative in the form of a Framework Convention on Alcohol Control, similar to that launched by the WHO for tobacco [93].

## Abbreviations

AUD: alcohol use disorder
HIC: high income countries
LMIC: low- and middle-income countries
RCT: randomized controlled trials
SBI: screening and brief intervention
